# Maternal region of birth and stillbirth in Victoria, Australia 2000–2011: A retrospective cohort study of Victorian perinatal data

**DOI:** 10.1371/journal.pone.0178727

**Published:** 2017-06-06

**Authors:** Miranda L. Davies-Tuck, Mary-Ann Davey, Euan M. Wallace

**Affiliations:** 1 The Ritchie Centre, Hudson Institute of Medical Research, Clayton, Melbourne, Victoria, Australia; 2 Department of Obstetrics and Gynaecology, School of Clinical Sciences, Monash University, Clayton, Melbourne, Victoria, Australia; Univesity of Iowa, UNITED STATES

## Abstract

**Background:**

There is growing evidence from high-income countries that maternal country of birth is a risk factor for stillbirth. We aimed to examine the association between maternal region of birth and stillbirth between 2000 and 2011 inclusive in Victoria, Australia.

**Methods:**

Retrospective population based cohort study of all singleton births at 24 or more weeks gestational age from 2000–2011 in Victoria, Australia. Stillbirths due to termination of pregnancy, babies with congenital anomalies and Indigenous mothers were excluded. Main Outcome Measure: Stillbirth.

**Results:**

Over the 12-year period there were 685,869 singleton births and 2299 stillbirths, giving an overall stillbirth rate of 3·4 per 1000 births. After adjustment for risk factors, compared to women born in Australia/New Zealand, women born in South Asia (aOR 1.27, 95% CI 1.01–1.53, p = 0.01), were more likely to have a stillbirth whereas women born in South East and East Asia were (aOR 0.60, (95% CI 0.49–0.72, p<0.001) less likely to have a stillbirth. Additionally, the increasing rate of stillbirth as gestation length progressed began to rise earlier and more steeply in the South Asian compared to Australian/New Zealand born women. The following risk factors were also significantly associated with an increased odds of stillbirth in multivariate analyses: maternal age <20 and 35 years and more, nulliparity, low socio-economic status, previous stillbirth, no ultrasound reported in 1^st^ trimester, pre-existing hypertension, antepartum haemorrhage and failure to detect growth restriction antenatally.

**Conclusion:**

Maternal region of birth is an independent risk factor for stillbirth. Improvements in the rate of stillbirth, particularly late pregnancy stillbirth, are likely to be gained in high-income settings where clinical care is informed by maternal region of birth.

## Introduction

There is still much to do in reducing the many preventable stillbirths that continue to occur in both high and low income countries[1–3]. Central to any effort in reducing the rate of stillbirth is a firm understanding of the key causes. Globally, the risk factors for stillbirth with the highest population attributable risks are advanced maternal age, maternal infections, non-communicable diseases, obesity, and prolonged pregnancy[3]. That many of these are increasing in prevalence [4] may explain, at least in part, why the rate of stillbirth is not decreasing despite advances in maternity care.

One risk factor for stillbirth in high-income countries (HIC), for which there is growing evidence is maternal country of birth. It is widely appreciated that stillbirths are relatively more common among women of certain ethnic groups. However, this apparently increased risk has been mostly discussed in the context of migration and social disadvantage rather than ethnicity *per se* [3, 5]. While both migration, particularly for humanitarian reasons[6], and social disadvantage are risk factors for stillbirth we believe that they may have obscured the influence of maternal region of birth itself. Maternal region of birth has been shown to be an independent risk factor for stillbirth in many high-income countries including the UK[7, 8], the Netherlands[9], Sweden[10], Singapore[11], and, Australia[12, 13]. Compared to locally born women, women of South Asian or African birth have a significantly higher rate of stillbirth while women of South East/East Asian birth have a significantly lower rate. The differences are not trivial. In an urban Australian population South Asian born women were nearly two and a half times more likely to have a late pregnancy stillbirth than their Australian born counterparts accessing the same public maternity services[13]. Similarly, in the UK African, Indian and, Pakistani women were more than twice as likely to have a stillbirth than white women[7]. For two consecutive years the UK Perinatal MBRRACE reports have shown that the rate of stillbirth is significantly higher among black and Asian women than among others[14, 15]. However, only the American Congress of Obstetricians and Gynecologists (ACOG) clinical guidelines recognise “black women” as being at increased risk of stillbirth. While clinical guidelines from other leading authorities, such as the Royal College of Obstetricians and Gynaecologists, the National Institute of Clinical Excellence, and the Royal Australian and New Zealand College of Obstetricians and Gynaecologists recognise maternal characteristics such as obesity, advanced maternal age, and, nulliparity as risk factors for stillbirth they are silent on maternal region of birth. With increasing migration to HIC the impact of maternal region of birth on overall rates of stillbirth is increasing. We believe that for the recent “re-call to action”^3^ to be successful an improved understanding of how maternal region of birth increases stillbirth risk will be needed. In this study we aimed to determine the rates of stillbirth in Victoria, Australia by maternal region of birth, exploring differences between different region of birth groups.

## Methods

We undertook a retrospective cohort study using data that are routinely reported on all births in Victoria, Australia to the government’s Victorian Perinatal Data Collection (VPDC) between 2000 to 2011. The authors had full access to the data. For every birth ≥ 20 weeks gestation (or ≥400g birth weight if gestation not known), regardless of place of birth the VPDC receives a standardised report detailing over 100 items regarding maternal characteristics, obstetric conditions, procedures and outcomes, perinatal mortality and morbidity and, birth defects. Validation of the accuracy of the dataset has been reported for two of the years included in this study– 2003 and 2011[16, 17]. The most recent validation assessed 93 variables in the dataset with 17 variables having 99% accuracy and 46 having 95% accuracy. Accuracy was below 80% for 9 items introduced in 2009. Agreement between medical records and the VPDC data ranged from 48% to 100%. In regards to the specific variables we used: maternal country of birth was 94% and stillbirth was 100% accurate. For all other variables assessed the accuracy ranged between 91% and 98% with the exception of maternal BMI (64%, smoking 77% and antenatal care provider (87%)[17]. There were no missing data for the outcome of stillbirth. For country of birth, 0.1% of data were missing. For the covariates, data were missing for 3.1% of the Index of Relative Socio-economic Disadvantage (IRSD–one of the area-level measures of socio-economic status derived from Australian 2011 census data), 0.002% for parity, 0.02% for maternal age, and 1.5% of previous caesarean section. Missing data were case-wise excluded. Where data were missing for ‘discipline of antenatal care provider’, ‘1^st^ trimester ultrasound’, ‘prior stillbirth’, ‘public/private admission status’, ‘smoking’ and, BMI a ‘not reported’ variable coding was created within the variable.

De-identified data for all singleton births ≥24 weeks gestational age in Victoria from 2000–2011 were extracted from the VPDC. Stillbirths due to termination of pregnancy, babies with congenital anomalies and Indigenous mothers were excluded. Indigenous mothers were excluded as it is well known, and widely published, that Indigenous women have significantly higher rates of stillbirth compared to non-Indigenous Australian born women. Accordingly, it was important that we specifically excluded them from the Australian/NZ born group to prevent bias.

The primary outcome was stillbirth, defined here as a fetal death at or after 24 weeks gestation.

Maternal ethnicity is not reported to the VPDC. Maternal region of birth was used for these analyses. Countries of birth were classified into regional groups, as defined by the United Nations[18], as a surrogate measure of ethnicity, according to an individual’s self-reported country of birth. Women were defined as being born in Australia or New Zealand (AUS/NZ), South Asia (SA) (India, Pakistan, Sri Lanka, Afghanistan and Bangladesh), South East and East Asia (SE-EA) (Vietnam, Malaysia, Indonesia, China, Japan), the Middle-east (ME) (Iraq, Israel, Joron, Turkey, Yemen, Cyprus), Africa, Europe or, ‘other’.

We also extracted relevant risk factors for stillbirth from the VPDC to allow adjusted analyses. Maternal medical conditions were identified by searching for the relevant ICD-10 AM codes. The following covariates were examined for all years: maternal age, parity (nulliparous or parous), IRSD quintiles, prior stillbirth (no prior birth, yes, no, not recorded), previous caesarean (previous caesarean, no previous caesarean, no prior birth), first trimester ultrasound as a marker of early engagement in antenatal care (yes, no, not recorded), private or public admission for the birth, pre-existing hypertension, pre-existing diabetes, pre-existing thyroid disease, gestational hypertension, gestational diabetes, preeclampsia/eclampsia/HELLP (Hemolysis (H), elevated liver enzymes (EL), and low platelet count (LP) occurring in pregnancy), antepartum haemorrhage, gestational age at birth (preterm (24 to 36^+6^ weeks), term (37^+0^ to 41^+6^ weeks) and post term (42 weeks plus)), baby sex, birth weight and, suspected growth restriction. Birth weights were plotted on a population based birth chart [19] and babies were defined as small for gestational age (SGA) if the birth weight was less than the 10^th^ centile for gestation and sex[20]. Previous work has reported differing stillbirth rates by whether fetal growth restriction was detected in pregnancy or not[7]. Therefore, we searched maternal medical conditions, obstetric complications and, indications for induction or operative delivery to identify where growth restriction had been identified using ICD-10-AM codes. We then computed a variable ‘detection of SGA’ where babies who were not SGA were compared to those who were SGA where growth restriction was detected and those who SGA where the growth restriction was not detected. From 2009 onwards the following additional variables were also available: body mass index (BMI) (<18.5, 18.5–24.99, 25–29.99,30–34.99, ≥35), smoking (non-smoker, quit by 20 weeks gestation, smoking at 20 weeks gestation and beyond or not recorded) and, lead antenatal care provider (obstetrician, midwife, general practitioner, none/not recorded).

### Statistical methods

Continuous variables were categorised as outlined above. Descriptive statistics were tabulated for each region of birth group. Cells within tables with fewer than 5 cases are reported as ‘<5’ as per VPDC standard rules. The rates of stillbirth per 1000 births for each region of birth group and by each gestational week were determined. The overall rate of stillbirth was calculated as the number of stillbirths divided by the total number of births after 24 weeks. The rate of stillbirth per week was calculated as the number of stillbirths in that gestational week divided by the total number of births from that week onwards[21]. The rate of stillbirth per 1000 births for each of the covariates was also determined. The distributions of gestation at birth for spontaneous labours (excluding labours that were induced and pre-labour caesarean sections) were graphed and the median gestation at onset of labour by region of birth compared using the Wilcoxon Rank Sum test. Logistic regression was performed to determine the association between maternal region of birth, risk factors for stillbirth and stillbirth. Co-variates were selected *a priori*. All variables significant at the univariate level (p<0.05) were included in the final regression model. Backward stepwise regression was performed. The likelihood ratio was used to determine the final model. Potential interactions between covariates and maternal country of birth on stillbirth were assessed by including interaction terms in the model. The rates of stillbirth differed significantly by gestational group therefore an analysis was also performed stratified by gestation. Because women could have had more than one birth during the period of the study, the resulting non-independence in the dataset was explored by also running all analyses in nulliparous women only. This did not alter the association between maternal region of birth and stillbirth. Due to the rare nature of the outcome we present the results for all women to allow multivariate analyses. A p-value<0.05 (two-tailed) was considered statistically significant. All analyses were performed using the SPSS statistical package version 20 (IBM Corp., Armonk, New York, USA).

### Ethics

This study was granted ethical approval by the Monash University Human Research Ethics Committee (MUHREC) CF12/2748_2012001499 1^st^ Nov 2012. The Consultative Council on Obstetric and Paediatric Mortality and Morbidity (CCOPMM) approved access to and analysis of the data.

### Data availability

The data used in this manuscript were obtained from a third party. The data were provided by the Clinical Councils Unit at the Victorian Government Department of Health and Human Services. Contact details for the owner of the data are: Email:clinical.councils@dhhs.vic.gov.au

## Results

Between 2000–2011 there were 685 869 singleton births at 24 or more completed weeks of gestation in Victoria, Australia. Seventy-four percent (n = 505 358) of the women giving birth were themselves born in either Australia or New Zealand (AUS/NZ), 3.7% (n = 25 634) in South Asia (SA), 8.0% (n = 54 714) in South-East or East Asia (SE-EA), 2.2% (n = 15 386) in the Middle East (ME), 2.5% (n = 17 098) in Africa, 5.8% (n = 39 576) in Europe (Eu) and, the remaining 4.0% (n = 28 103) were born elsewhere. The characteristics of the women are presented in Table 1.

**Table 1:** Characteristics of population (> = 24 weeks, singleton, non-Indigenous, no congenital anomaly and excluding terminations) by maternal region of birth.

	**Australia N = 505358**	**South Asia N = 25634**	**South East-East Asia N = 54714**	**Middle East N = 15386**	**Africa N = 17098**	**Europe N = 39576**	**Other N = 28103**
**2000–2011**	n(%)	n(%)	n(%)	n(%)	n(%)	n(%)	n(%)
Maternal Age							
*Younger than 20yrs*	16469(3)	179(0.7)	779(1)	543(4)	461(3)	530(1)	926(3)
*20–34 yrs*	365534(72)	19700(77)	38683(71)	11509(75)	12272(72)	25214(64)	19182(68)
*35plus yrs*	123250(24)	5746(22)	15248(28)	3333(22)	4361(26)	13828(35)	7990(28)
Primiparous	215393(43)	13604(53)	25628(49)	4628(30)	6023(35)	16512(42)	11079(62)
	**Australia N = 505358**	**South Asia N = 25634**	**South East-East Asia N = 54714**	**Middle East N = 15386**	**Africa N = 17098**	**Europe N = 39576**	**Other N = 28103**
IRSD quintiles							
*1-Most disadvantaged*	79211(16)	7320(30)	16706(31)	7956(53)	5481(33)	5877(15)	5799(21)
*2*	100689(22)	4836(20)	9149(17)	2322(15)	3450(21)	5603(14)	5193(19)
*3*	103976(21)	4732(19)	9471(18)	2058(14)	2742(17)	8083(21)	5745(21)
*4*	104187(21)	3790(16)	7780(15)	1335(9)	2250(14)	8760(23)	5115(19)
*5-Least disadvantaged*	100946(22)	3695(15)	10447(20)	1319(9)	2740(16)	10491(27)	5536(20)
Previous stillbirth	5583(1)	376(2)	438(1)	314(2)	71(0.4)	452(1)	331(1)
Previous caesarean delivery	71978(15)	3716(15)	6430(12)	1997(13)	2659(16)	5319(14)	3717(14)
	**Australia N = 505358**	**South Asia N = 25634**	**South East-East Asia N = 54714**	**Middle East N = 15386**	**Africa N = 17098**	**Europe N = 39576**	**Other N = 28103**
First trimester Ultrasound							
*No*	129730(26)	9405(37)	23497(43)	9433(61)	8499(50)	11279(29)	10726(38)
*Not recorded*	12539(3)	354(1)	1715(3)	297(2)	513(3)	957(2)	726(3)
Private patient	177271(35)	3964(16)	11439(21)	1690(11)	3101(18)	13356(34)	6817(24)
Pre-existing Hypertension	6677(1)	149(0.6)	305(0.6)	90(0.6)	139(1)	390(1)	305(1)
Pre-existing Diabetes	5961(1)	1327(5)	1606(3)	328(2.1)	386(2)	517(1)	458(2)
Pre-existing Thyroid disease	4665(1)	409(2)	577(1)	138(1)	152(1)	435(1)	234(1)
Gestational Hypertension	15759(3)	576(2)	888(2)	283(2)	368(2)	978(3)	780(3)
	**Australia N = 505358**	**South Asia N = 25634**	**South East-East Asia N = 54714**	**Middle East N = 15386**	**Africa N = 17098**	**Europe N = 39576**	**Other N = 28103**
Gestational Diabetes	18385(4)	2990(12)	5973(11)	1125(7)	1063(6)	2014(5)	1424(5)
Pre eclampsia/HELLP	14784(3)	537(2)	949(2)	268(2)	400(2)	1028(3)	758(3)
APH	16624(3)	670(3)	2300(4)	425(3)	399(2)	1210(3)	839(3)
Suspected FGR	11892(2)	967(4)	1200(2)	397(3)	383(2)	767(2)	548(2)
Gestational age (completed weeks)							
*24–36 weeks*	28534(6)	1436(6)	2930(5)	728(5)	907(5)	2075(5)	1547(6)
*37–41 weeks*	470004(93)	23948(93)	51377(93)	14469(94)	15743(92)	37001(94)	24564(89)
*42weeks+*	6820(1)	250(1)	407(0.7)	189(1)	448(3)	500(1)	444(2)
	**Australia N = 505358**	**South Asia N = 25634**	**South East-East Asia N = 54714**	**Middle East N = 15386**	**Africa N = 17098**	**Europe N = 39576**	**Other N = 28103**
Birthweight							
*Under 10*^*th*^ *Centile*	40531(8)	4612(18)	6811(12)	1505(10)	1839(11)	2975(8)	2140(8)
*Under 3*^*rd*^ *Centile*	11640(2)	1480(6)	1695(3)	393(3)	517(3)	823(2)	597(2)
Baby sex(Male)	257367(51)	13127(51)	27967(51)	7832(51)	8709(51)	20245(51)	14430(51)
**2009–2011 only**	**N = 90812**	**N = 10209**	**N = 12534**	**N = 2965**	**N = 3934**	**N = 6587**	**N = 6452**
Body Mass Index							
*<18*.*5*	1920(2)	413(4)	854(7)	39(1)	113(3)	150(2)	97(2)
*18*.*5–24*.*99*	39094(43)	4792(57)	7443(59)	1072(36)	1408(49)	3217(49)	2390(37)
	**Australia N = 90812**	**South Asia N = 10209**	**South East-East Asia N = 12534**	**Middle East N = 2965**	**Africa N = 3934**	**Europe N = 6587**	**Other N = 6452**
*25–29*.*99*	22288(26)	2486(24)	1700(14)	721(24)	894(22)	1461(22)	1504(23)
*30–34*.*99*	10800(12)	6716(7)	383(3)	294(10)	368(7)	481(7)	898(14)
*35–39*.*99*	4715(5)	124(1)	55(0.4)	118(4)	101(2)	154(2)	447(7)
*40plus*	2658(3)	34(0.3)	19(0.2)	43(2)	37(1)	62(1)	299(5)
Smoking							
*Non-smoker*	76559(84)	10103(99)	12131(97)	2742(93)	3758(96)	5968(91)	5443(84)
*Quit by 20 weeks*	2319(3)	34(0.3)	120(1)	49(2)	50(1)	186(3)	209(3)
*Smoking at 20 weeks*	10394(11)	27(0.3)	155(1)	152(5)	87(2)	356(5)	676(11)
*Not stated*	1540(2)	45(0.4)	128(1)	22(0.7)	39(1)	77(1)	124(2)
	**Australia N = 90812**	**South Asia N = 10209**	**South East-East Asia N = 12534**	**Middle East N = 2965**	**Africa N = 3934**	**Europe N = 6587**	**Other N = 6452**
Antenatal Care Provider							
*Obstetrician*	50314(55.4)	4298(42.1)	5683(45.3)	1456(49.1)	1717(43.6)	3467(52.6)	3060(47.4)
*Midwife*	23776(26.2)	3965(38.8)	5071(40.5)	1129(38.1)	1631(41.5)	2010(30.5)	2196(34.0)
*GP*	15548(17.1)	1864(18.3)	1659(13.2)	359(12.1)	539(13.7)	1058(16.1)	1106(17.1)
*None*	312(0.3)	39(0.4)	55(0.4)	16(0.5)	28(0.7)	16(0.3)	50(0.8)

Number (Percentage)

NB: Numbers may not add up to total number due to missing data as outlined in the methods section.

### Maternal region of birth and stillbirth

There were a total of 2299 stillbirths giving an overall stillbirth rate of 3.4 per 1000 births. About 60% of those occurred before 37 weeks gestation. The rates of stillbirth differed by maternal region of birth. Overall the rate of stillbirth in AUS/NZ born women was 3.3 per 1000 (n = 1669), compared to 5.1 per 1000 in SA (n = 131), 2.4 per 1000 in SE-EA (n = 133), 4.4 per 1000 in ME (n = 67) and African (n = 76), 3.5 per 1000 in Eu women (n = 138) and, 3.0 per 1000 in ‘other’ born women (n = 85). After adjustment for risk factors significant at the univariate level, compared to women born in AUS/NZ those born in SA were 27% (95% CI 1.01 to 1.53, p = 0.01) more likely to have a stillbirth while women born in SE-EA were 40% (95% CI 0.49 to 0.72, p<0.001) less likely to have a stillbirth (Table 2). There was a much higher rate of stillbirth in preterm births (35.9 per 1000) compared to term (1.4 per 1000) and post term (1.0 per 1000) births and there was evidence for an interaction between gestation and maternal region of birth (p<0.001) on stillbirth. Therefore, we also stratified the analyses by gestation (Table 2). South Asian region of birth was more strongly associated with a term/post-term stillbirth than a preterm stillbirth. In contrast, SE-EA region of birth was most strongly protective in the term/post-term period while ME region of birth was only significantly associated with preterm stillbirths. Other risk factors for stillbirth from the univariate and multivariate analyses are presented in S1 and S2 Tables respectively. Based on a report from the UK^7^ we also examined whether being born below the 10^th^ centile and antenatal suspicion of growth restriction were associated with stillbirth in our population. We observed that where SGA existed and had been detected there was a 2.3 fold increased odds (95% CI 1.82 to 3.04, p<0.001) of stillbirth. However, in those pregnancies where an SGA baby had not been identified before birth the odds of stillbirth was increased 4.3-fold (95% CI 3.92 to 4.76; p<0.001) (S2 Table).

**Table 2:** Adjusted association between maternal region of birth and stillbirth for all gestation, pre-term and term birth.

	Number of Stillbirths	All gestations Adjusted Odds Ratio (95%CI) ^1^	P value	Preterm Adjusted Odds Ratio (95%CI) ^2^	P value	Term Adjusted Odds Ratio (95%CI) ^3^	P value
Maternal Region of Birth							
Australia/NZ	1669	Reference	-	Reference	-	Reference	-
South Asia	131	1.27(1.01 to 1.53)	**0.01**	1.33(1.02 to 1.74)	**0.04**	1.54(1.17 to 2.0)	**0.002**
South-East-East Asia	133	0.60(0.49 to 0.72)	**<0.001**	0.81(0.64 to 1.03)	0.08	0.55(0.41 to 0.74)	**<0.001**
Middle East	67	1.14(0.88 to 1.47)	0.33	1.47(1.04 to 2.08)	**0.03**	1.27(0.86 to 1.88)	0.24
Africa	76	1.21(0.95 to 1.53)	0.12	1.35(0.98 to 1.87)	0.07	1.33(0.93 to 1.92)	0.21
Europe	138	1.09(0.91 to 1.30)	0.34	1.26(0.99 to 1.60)	0.06	0.97(0.73 to 1.30)	0.86
Other	85	0.87(0.69 to 1.30)	0.22	1.04(0.78 to 1.40)	0.77	0.72(0.49 to 1.07)	0.72

^**1**^Odds Ratio of Stillbirth by maternal region of birth compared to Australia/NZ region of birth adjusted for maternal age, IRSD, parity, 1^st^ trimester ultrasound, pre-existing hypertension, gestational hypertension, APH, detection of SGA, previous stillbirth, GDM and PE/HELLP.

^**2**^Odds Ratio of Preterm Stillbirth by maternal region of birth compared to Australia/NZ region of birth adjusted for maternal age, IRSD, 1^st^ trimester ultrasound, previous stillbirth, gestational hypertension, GDM, PE/HELLP, APH and SGA detection.

^3^Odds Ratio of Term Stillbirth by maternal region of birth compared to Australia/NZ region of birth adjusted for maternal age, parity, IRSD, 1^st^ trimester ultrasound, pre-existing hypertension, gestational hypertension, APH, and SGA detection.

**Fig 1:**
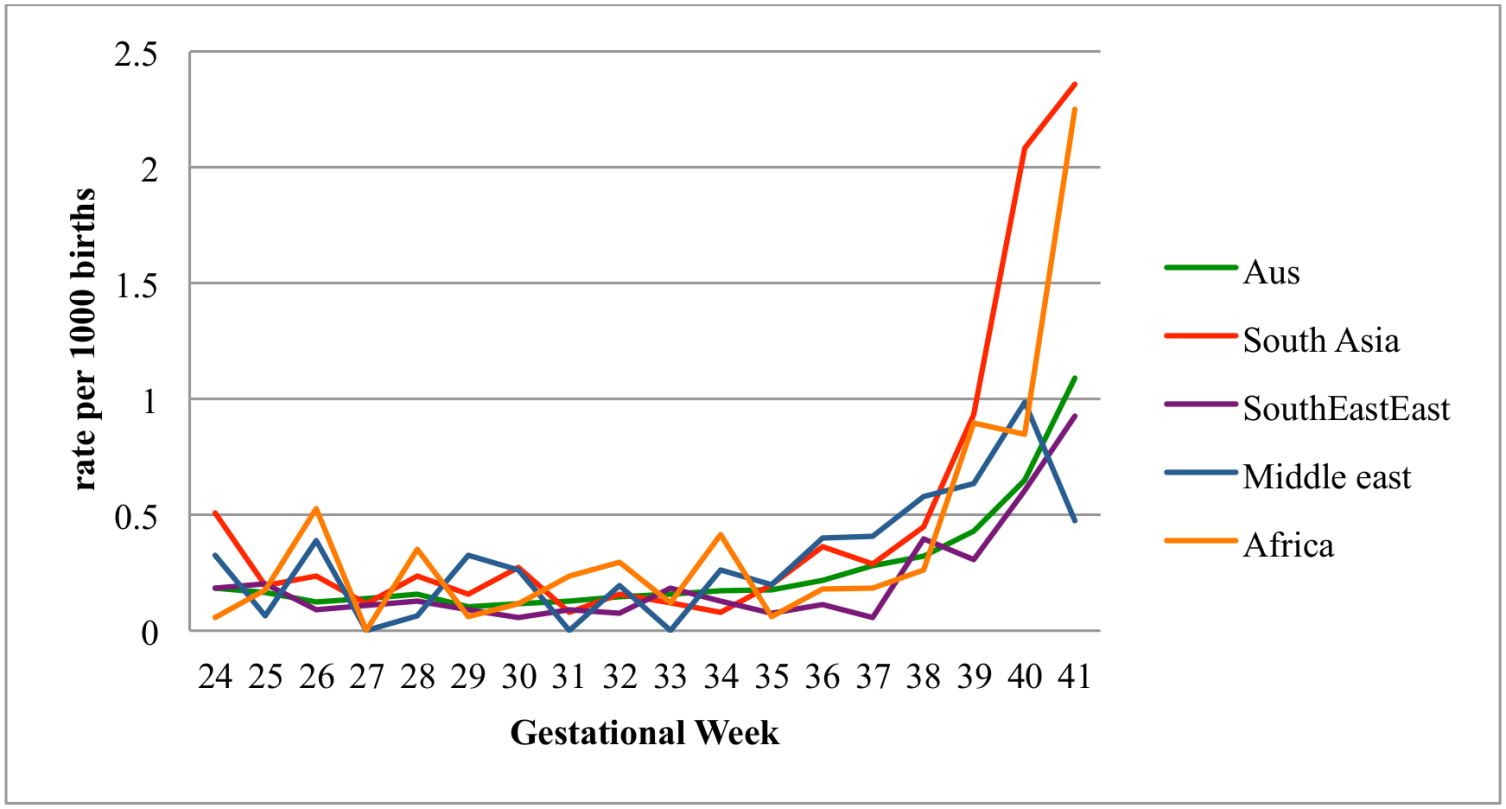
The rate of stillbirth per 1000 births for each gestational week by region of birth.

The specific rate of stillbirth by region of birth and gestational week is presented in Fig 1 The rate of stillbirth for all groups remained fairly stable from 24–37 weeks gestation. After 37 weeks gestation the rate of stillbirth in SA and ME born women was higher than the rate in AUZ/NZ born women. After 38 weeks gestation the stillbirth rate was also higher in African born women than AUZ/NZ born women while the rate in SE-EA born women was similar to that in AUS/NZ born women. The rate of stillbirth in SA and African born women at 39 weeks was similar to that of AUS/NZ born women at 41 weeks. The specific relative rates of stillbirth per 1000 on-going pregnancies by country of birth and gestation week from 37 weeks are presented in Table 3.

**Table 3:** Relative Rate of stillbirth per 1000 ongoing pregnancies per week compared to AUS/NZ women from 37 weeks gestation.

	South Asia	South-East-East Asia	Middle East	Africa
37 weeks	1.03	0.21	1.46	0.66
38 weeks	1.39	1.23	1.79	0.81
39 weeks	2.17	0.72	1.47	2.08
40 weeks	3.19	0.93	1.51	1.30
41 weeks and beyond	2.16	0.84	0.43	2.07

**Fig 2:**
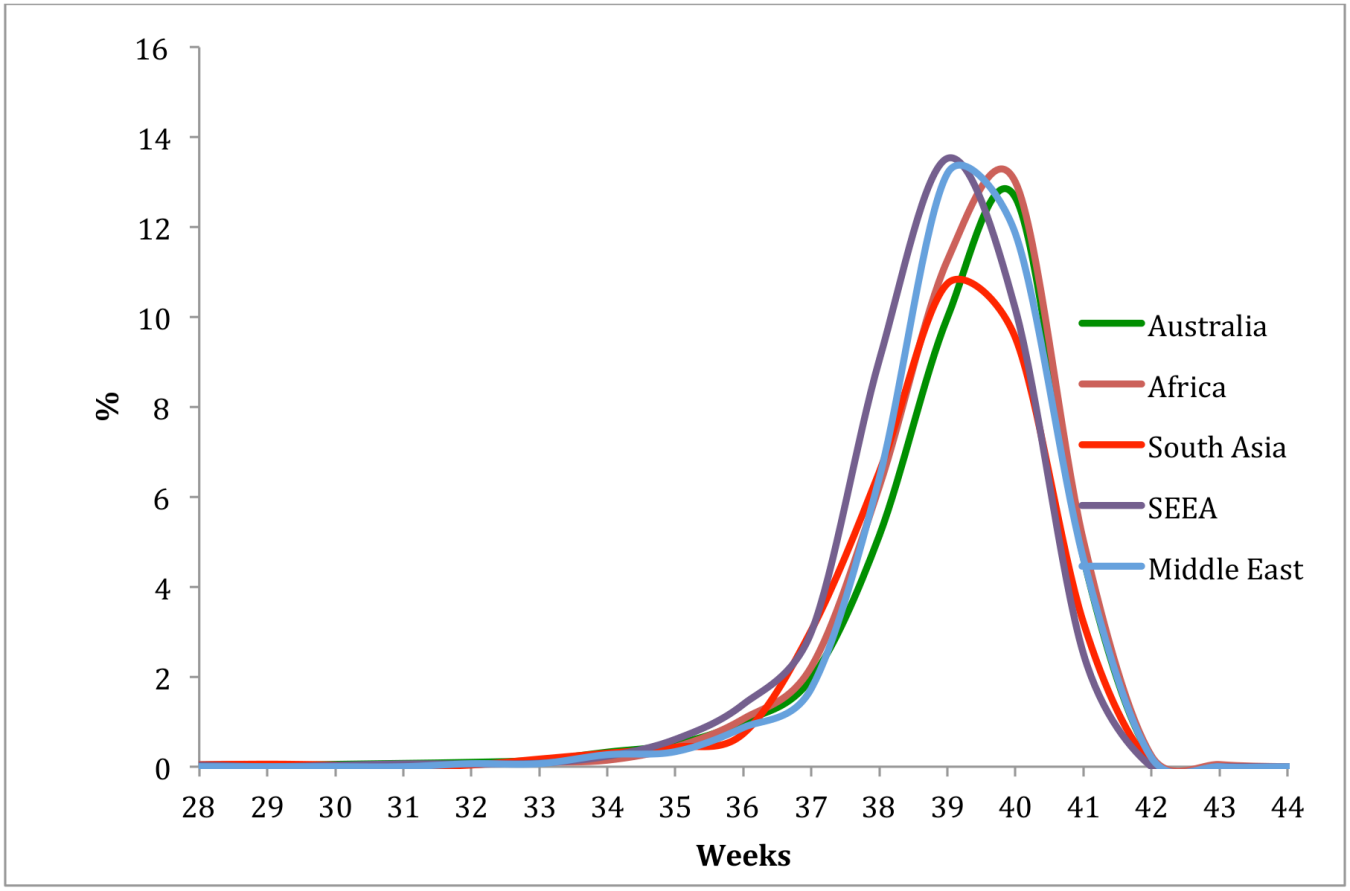
Frequency distributions of spontaneous onset of labour by region of birth.

Finally, because of the earlier increase in the rate of term stillbirths in SA women we wondered whether there were ethnic differences in the timing of feto-placental maturation, and therefore post-maturity. It has been suggested previously that there may be gestational length differences among women of different ethnicities.[19] Fig 2 presents the distributions of spontaneous onset of labour by maternal country of birth. Overall the median onset of labour for AUS/NZ born women was 40 weeks, for SA born women it was 39 weeks, for SE-EA born women it was 39 weeks, for African born women it was 40 weeks and, for ME born women it was 40 weeks (p<0.001).

## Discussion

We have confirmed other reports that there is an association between maternal region of birth and stillbirth. Compared to AUS/NZ born women, women born in SA, Africa and the ME have higher rates of stillbirth while women born in SE-EA had a lower rate. There was also evidence for an interaction between maternal region of birth and the gestation at which stillbirths occur. Women born in SA had a stronger odds of term compared to preterm stillbirth whereas women born in the ME had an increased odds of preterm stillbirth only. Regarding late pregnancy stillbirths, the rate of stillbirth increased earlier in pregnancy, and rose more rapidly in SA and African born women compared to AUS/NZ born women. These observations have direct implications for the care of women during pregnancy and approaches to reduce the burden of stillbirth.

Disparities in the rates of stillbirth among migrant women have been previously reported in several high-income countries [7, 9, 13, 22]. However, this increased risk has usually been discussed in relation to factors related to migration such as social deprivation, lack of education, troubles communicating and understanding advice and poor access to care[3]. These are certainly important. In the current study we have confirmed disparities in social deprivation and access to pregnancy care by maternal region of birth. However, that the odds of stillbirth after adjustment for these confounding factors remained higher among SA, African, and, ME born women and lower in SE-EA born women suggests that maternal region of birth is, in itself, an important risk factor. In particular, the odds ratio for term stillbirth in SA born women, at 1.5, is broadly the same as that for smoking, preeclampsia or obesity[3]. That maternal region of birth itself is an independent risk factor is particularly supported by our observation that SE-EA born women, who despite having increased prevalence of social disadvantage, poor access to 1^st^ trimester ultrasound, pre-existing diabetes, GDM, APH and, small for gestation babies had the lowest rates of stillbirth, with almost half the rate of term stillbirth than AUS/NZ born women and a third of the rate of SA or African born women.

We also identified that the association between maternal region of birth and stillbirth differed significantly by gestation. ME region of birth was only associated with preterm stillbirths. The explanation for this is not immediately apparent but it may reflect a higher rate of consanguinity [23] in this group which has been shown to be associated with preterm birth[24]. In contrast, SA region of birth was more strongly associated with late pregnancy stillbirth and the rate of stillbirth increased more steeply and at an earlier gestation in south Asian and African born women. These findings are consistent with other studies that determined maternal region of birth specific differences in perinatal mortality at different gestational ages[9, 25].

We also found, consistent with others[26, 27], that the median gestation at which spontaneous labour onset occurred was significantly earlier in SA born women. It is generally agreed that at term and beyond the placenta is progressively unable to meet the increasing metabolic needs of the fetus, leading to falling fetal oxygenation[28] and this explains the increased risk of stillbirth in the post term period. Our findings suggest that there may be maternal region of birth differences in the rate of “placental aging”. Supporting this, Indian women have significantly smaller placental surface area, weight and volume than Malaysian and Chinese women after correction for gestation of baby and maternal size[29]. Differences in other aspects of placental function may therefore also exist that explain the shorter average duration of pregnancy and the higher stillbirth rates in late pregnancy. Finally there is emerging evidence to suggest that telomeres and the enzyme that controls their length, telomerase differ by ethnicity and are associated with stillbirth and intra-uterine growth restriction[28]. Intriguingly, we also observed that SE-EA born women also had shorter pregnancies than AUS/NZ and Eu women and yet they had the lowest stillbirth rates of all women. Further research aimed at understanding why this group of women have the lowest rates of stillbirth is needed. Other factors that may explain the increased rate of stillbirth in these region of birth groups that were not able to be assessed in this study include time spent in Australia, cultural practices and language barriers. Further work aimed at understanding all of these mechanisms is needed. Nonetheless, as highlighted in the most recent Lancet Stillbirth series 14% of stillbirths worldwide are attributed to a prolonged pregnancy[3]. Whatever the mechanism(s), our findings, and those of others [9, 25, 30], suggest that the optimum gestation for birth may differ by a mother’s region of birth. Of course, what defines optimal birth gestation for women of different backgrounds is unclear. A population based study in the Netherlands aimed to answer this very question[31]. Although though not statistically significant, otherwise healthy African women tended to have a lower risk of adverse outcomes from 38 weeks gestation with an induced earlier birth rather than expectant management. Not surprisingly, the numbers of induced births needed to prevent one stillbirth were very large [31] suggesting that routine induction of labour is unlikely to be a suitable approach. In our current study, at 39 weeks gestation SA and African born women had a rate of stillbirth similar to AUS/NZ born women at 41 weeks. Clinical care guidelines (NICE guidelines, Royal Australia and New Zealand College of Obstetricians and, Gynaecologists Guidelines (RANZCOG)) recommend routine ultrasound assessment and/or induction of pregnancies that are post-term. A more pragmatic approach to reduce the rates of stillbirth in these groups may be to suggest that post-term surveillance in south Asian and African born women begins at 39 weeks[9, 25, 30].

Being small for gestational age is the largest single risk factor for stillbirth with over half of all “unexplained” stillbirths being SGA[7]. If this is true for our population then undetected fetal growth restriction could explain up to 1/3 of all Victorian stillbirths. We would echo the call of others [32, 33] that the better detection of the growth restricted fetus should be a focus of improvements in clinical care provision. Indeed, regardless of maternal region of birth, we found that where growth restriction remained undetected there was a 4.3 fold increased odds of stillbirth. This is consistent with a recent UK study that reported a 6.5 fold increased risk of stillbirth where growth restriction was undetected[7]. However, antenatal detection of the growth restricted fetus, particularly at term, remains challenging[34]. In our study only 29% of babies born to AUS/NZ born mothers who were born SGA were detected antenatally. In babies born to SA and African born mothers it was only 21%. This is particularly relevant as the frequencies of SGA babies are higher in Asian and African born women. While improving detection is an obvious target, how best to achieve this is less clear[34]. There is growing interest in the application of antenatal growth charts customized for maternal height, weight, parity, ethnicity and, fetal sex to improve the detection of SGA babies[32]. Critics of customization however argue that the influence of these factors, in particular maternal ethnicity cannot be considered a physiological driver of baby size[35]. SA and African immigrant women have both higher rates of SGA infants and stillbirth, so customizing for maternal ethnicity may lead to false reassurance of appropriate fetal growth and consequently increase the rate of stillbirth in these groups. Caution is therefore advised in regards to customizing with maternal region of birth. Further work is required to identify the most appropriate approach to detect SGA babies in women of Asian and African descent.

Our study had a number of limitations. Maternal “ethnicity” was defined as self reported region of birth. It is possible that some women who were classified as Australian were in fact of other ethnicities. This is a weakness of perinatal data in most jurisdictions in Australia that do not explicitly collect ethnicity information. Data from the Australian Bureau of Statistics (2013) states that among Australian born individuals (men and women), the most common reported ancestries are English (36%), Australian (35%), Irish (10%) and Scottish (9%). Chinese and Indian ancestry was identified in 4% and 2% respectively. As such the findings of this study can only inform the risk of stillbirth relative to where the mother was born, not her ancestry/ethnicity. Secondly, due to the rare nature of stillbirth, we may have been underpowered to assess risk factors for stillbirth stratified by term and preterm. However, a number of significant risk factors for stillbirth were identified. Furthermore, the variables BMI, lead antenatal care provider and, smoking were only available from 2009 onwards. A lack of significant association may therefore reflect a lack of power rather than lack of association. In addition the timing of the stillbirth (antepartum or intrapartum) was also not reported until 2009 and as such we were unable to assess whether there were differences in when the stillbirth occurred relative to the onset of labour by maternal country of birth. Another limitation of our study is that we do not have the gestation at death, just the gestation of the delivery. Some babies may have died a number of days prior. Socio-economic status was defined at the area level, not at the individual level. It is possible that confounding from socioeconomic status still exists between the association between maternal country of birth and stillbirth. Fields were predominantly complete therefore missing data was not a big problem in this study. That said the identification of babies who were suspected as having growth restriction during pregnancy involved searching for obstetric complications, indications for induction and operative birth as well as obstetric procedures for IUGR ICD-10 codes. This relies on the clinicians including this information in the free text component of the perinatal data form. It is possible that some of the babies identified as undetected were in fact detected but not reported as such. If this were the case, it would have diluted the true association between undetected SGA and stillbirth, so our strong result would be an underestimate. An important consideration when interpreting our findings is that determination of gestational age at delivery relies on accurate menstrual dates or 1^st^ trimester dating, preferably with an early ultrasound. Rates of access to first trimester ultrasound were lower in all region of birth groups except “Europe”. It is possible that this may result in misclassification of the gestation of the birth. Finally, non-independence is a recognised issue when working with large perinatal datasets, therefore we ran all analyses in nulliparous women only. Doing so did not change the associations that we observed. Due to the rare nature of the outcome we presented data for all parities to preserve power. Strengths of our study include the use of a complete population-based dataset that has been found to have highly accurate data on the variables of interest here.

In conclusion, we identified that maternal SA and ME region of birth were independent risk factors for stillbirth. The rate of stillbirth also increased earlier in SA and African born women compared to AUS/NZ born women at term. Specifically, SA and African born women at 39 weeks had a rate of stillbirth similar to AUS/NZ born women at 41 weeks. Earlier post-term surveillance of SA born women and an emphasis on detection of fetal growth restriction may therefore be recommended. The call to include maternal country or region of birth in clinical guidelines has been made previously[36]. Our study provides further evidence that it is now time for all clinical guidelines to recognize maternal region of birth as a risk factor for stillbirth in its own right.

## Supporting information

S1 Table Crude association between maternal region of birth, covariates and stillbirth. (DOCX)

S2 Table Adjusted associations between covariates and stillbirth. (DOCX)
