# Supplementary material for: Maternal region of birth and stillbirth in Victoria, Australia 2000–2011: A retrospective cohort study of Victorian perinatal data
Source: PLoS One. 2017 Jun 6;12(6):e0178727. doi: 10.1371/journal.pone.0178727 (PMC5460852; doi:10.1371/journal.pone.0178727)
Supplement: S1 Table — (DOCX) [file pone.0178727.s001.docx]

**Supplementary Table 1**

**Crude association between maternal region of birth, covariates and stillbirth.**

|  | **Rate of stillbirth per 1000** | **Unadjusted Odds Ratio (95%CI)^1^** | **P Value** |
| --- | --- | --- | --- |
| 2000-2011  Maternal Country of Birth |  |  |  |
| *Australia* | 3.3 | Reference | - |
| *South Asia* | 5.1 | 1.6(1.3 to 1.9) | **<0.001** |
| *South-East-East Asia* | 2.4 | 0.7(0.6 to 0.9) | **0**.**001** |
| *Middle East* | 4.3 | 1.3(1.0 to 1.7) | **0**.**03** |
| *Africa* | 4.4 | 1.4(1.1 to 1.7) | **0**.**01** |
| *Europe* | 3.5 | 1.1(0.9 to 1.3) | 0.54 |
| *Other* | 3.0 | 0.9(0.7 to 1.1) | 0.43 |
| Maternal Age |  |  |  |
| *Younger than 20yrs* | 5.5 | 1.72(1.42 to 2.1) | **<0**.**001** |
| *20-34 yrs* | 3.2 | Reference | *-* |
| *35plus yrs* | 3.5 | 1.1(0.98 to 1.12) | 0.1 |
| Primiparous | 3.9 | 1.31(1.21 to 1.42) | **<0**.**001** |
| IRSD quintiles |  |  |  |
| *1-Lowest* | 4.3 | 1.67(1.46 to 1.91) | **<0**.**001** |
| *2* | 3.8 | 1.46(1.28 to 1.68) | **<0**.**001** |
| *3* | 2.9 | 1.14(0.99 to 0.13) | 0.07 |
| *4* | 3.1 | 1.19(1.03 to 1.37) | **0**.**02** |
| *5-Highest* | 2.6 | Reference | - |
| Previous stillbirth |  |  |  |
| *Yes* | 9.7 | 2.97(2.36 to 3.74) | **<0**.**001** |
| *No* | 3.3 | Reference | **-** |
| Previous caesarean delivery |  |  |  |
| *No previous pregnancy* | 3.8 | Reference | - |
| *No* | 3.1 | 0.82(0.75 to 0.90) | **<0**.**001** |
| *Yes* | 3.2 | 0.84(0.74 to 0.96) | **0**.**01** |
| First trimester Ultrasound |  |  |  |
| *Yes* | 2.9 | Reference | - |
| *No* | 4.2 | 1.43(1.31 to 1.56) | **<0**.**001** |
| *Not recorded* | 5.1 | 1.74(1.40 to 2.16) | **<0**.**001** |
| Admission Type |  |  |  |
| *Private patient* | 2.5 | 0.66(0.60 to 0.72) | **<0**.**001** |
| *Public patient* | 3.8 | Reference | **-** |
| Pre-existing Hypertension | 7.2 | 2.18(1.68 to 2.84) | **<0**.**001** |
| Pre-existing Diabetes | 4.6 | 1.39(1.04 to 1.84) | **0**.**02** |
| Pre-existing Thyroid disease | 3.5 | 1.04(0.69 to 1.57) | 0.86 |
| Gestational Hypertension | 2.4 | 0.72(0.54 to 0.96) | **0**.**03** |
| Gestational Diabetes | 3.4 | 0.79(0.63 to 0.97) | **0**.**03** |
| Pre eclampsia/HELLP | 6.3 | 1.95(1.62 to 2.34) | **<0**.**001** |
| APH | 14.6 | 4.96(4.41 to 5.58) | **<0**.**001** |
| Suspected IUGR | 5.9 | 1.81(1.47 to 2.22) | **<0**.**001** |
| Birthweight under 10^th^ Centile | 10.9 | 4.25(3.9 to 4.7) | **<0**.**001** |
|  |  |  |  |
| **2009-2011 only** |  | - | **-** |
| Body Mass Index |  | Reference |  |
| *<18.5* | 3.6 | 1.46(0.93 to 2.57) | 0.19 |
| *18.5-24.99* | 2.5 | Reference | **-** |
| *25-29.99* | 3.3 | 1.35(1.05 to 1.73) | **0**.**02** |
| *30-34.99* | 4.2 | 1.68(1.24 to 2.28) | **<0**.**001** |
| *35-39.99* | 3.8 | 1.55(0.99 to 2.43) | *0*.*06* |
| *40plus* | 4.7 | 1.92(1.12 to 3.26) | **0**.**02** |
| Smoking |  |  |  |
| *Non-smoker* | 3.1 | Reference | **-** |
| *Quit by 20 weeks* | 2.7 | 0.88(0.43 to 1.77) | 0.71 |
| *Smoking at 20 weeks* | 4.8 | 1.57(1.19to 2.08) | **0**.**002** |
| *Not stated* | 6.5 | 2.13(1.22 to 3.71) | **0**.**008** |
| Antenatal Care Provider |  |  |  |
| *Obstetrician* | 3.5 | Reference | **-** |
| *Midwife* | 2.8 | 0.79(0.63 to 0.99) | **0**.**04** |
| *GP* | 2.7 | 0.75(0.56 to 0.99) | **0**.**048** |
| *None* | 15.3 | 4.36(2.15 to 8.9) | **<0**.**001** |

^1^Odds ratio of stillbirth per respective variable
