# Supplementary material for: Maternal region of birth and stillbirth in Victoria, Australia 2000–2011: A retrospective cohort study of Victorian perinatal data
Source: PLoS One. 2017 Jun 6;12(6):e0178727. doi: 10.1371/journal.pone.0178727 (PMC5460852; doi:10.1371/journal.pone.0178727)
Supplement: S2 Table — (DOCX) [file pone.0178727.s002.docx]

**Supplementary Table 2: Adjusted associations between covariates and stillbirth**

|  | **All gestations Adjusted Odds Ratio (95%CI)** | **P value** | **Preterm**  **Adjusted Odds Ratio (95%CI)** | **P value** | **Term Adjusted Odds Ratio (95%CI)** | **P value** |
| --- | --- | --- | --- | --- | --- | --- |
| **2000-2011**  Maternal Age |  |  |  |  |  |  |
| *Younger than 20yrs* | 1.34(1.08 to 1.65) | **0.007** | 1.36(1.06 to 1.77) | **0**.**02** | 0.85(0.57 to 1.27) | 0.43 |
| *20-34 yrs* | Reference | *-* | Reference | - | Reference | - |
| *35plus yrs* | 1.20(1.09 to 1.33) | **<0**.**001** | 1.08(0.95 to 1.23) | 0.26 | 1.9(1.02 to 1.39) | **0**.**03** |
| Nulliparous | 1.25(1.14 to 1.36) | **<0**.**001** | NI | - | 1.51(1.34 to 1.71) | **<0**.**001** |
| IRSD quintiles |  |  |  |  |  |  |
| *1-Most disadvantaged* | 1.42(1.24 to 1.64) | **<0**.**001** | 1.26(1.05 to1.52) | **0**.**02** | 1.36(1.09 to 1.70) | **0**.**006** |
| *2* | 1.31(1.14 to 1.51) | **<0**.**001** | 1.11(0.92 to 1.35) | 0.27 | 1.39(1.12 to 1.73) | **0**.**003** |
| *3* | 1.07(0.92 to 1.23) | 0.39 | 0.97(0.80 to 1.18) | 0.77 | 1.10(0.88 to 1.37) | 0.42 |
| *4* | 1.15(0.99 to 1.33) | 0.06 | 1.05(0.86 to 1.27) | 0.65 | 1.12(0.96 to 1.49) | 0.11 |
| *5-Least disadvantaged* | Reference | - | Reference | - | Reference | - |
| Previous stillbirth | 2.9(2.27 to 3.70) | **<0**.**001** | 1.55(1.18 to 2.03) | **0**.**002** | NI | - |
| First trimester Ultrasound |  |  |  |  |  |  |
| *Yes* | Reference | - | Reference | - | Reference | - |
| *No* | 1.34(1.22 to 1.47) | **<0**.**001** | 1.26(1.11 to 1.42) | **<0**.**001** | 1.16(1.005 to 1.35) | **0**.**04** |
| *Not recorded* | 1.64(1.31 to 2.1) | **<0**.**001** | 1.56(1.14 to 2.13) | **0**.**005** | 1.61(1.13 to 2.29) | **0**.**007** |
| Pre-existing Hypertension | 2.0(1.52 to 2.62) | **<0**.**001** | NI | - | 2.06(1.32 to 3.22) | **0**.**001** |
| Gestational Hypertension | 0.64(0.47 to 0.89) | **0**.**002** | 0.56(0.37 to 0.84) | **0**.**005** | 0.64(0.40 to 1.03) | 0.06 |
| Gestational Diabetes | 0.68(0.53 to 0.86) | **0**.**001** | 0.47(0.34 to 0v64) | **<0**.**001** | NI | - |
| Pre eclampsia/HELLP | 1.59(1.31 to 1.93) | **<0**.**001** | 0.35(0.28 to 0.44) | **<0**.**001** | NI | - |
| APH | 14.98(1.98 to 2.29) | **<0**.**001** | 1.24(1.07 to 1.44) | **0**.**005** | 3.8(3.05 to 4.81) | **<0**.**001** |
| Detection of SGA |  |  |  |  |  |  |
| *Not SGA* | Reference | - | Reference | - | Reference | - |
| *SGA detected antenatal* | 2.35(1.82 to 3.04) | **<0.001** | 1.27(0.96 to 1.69) | 0.09 | 1.04(0.55 to 1.94) | 0.92 |
| *Undetected* | 4.32(3.92 to 4.76) | **<0**.**001** | 8.25(7.22 to 9.42) | **<0**.**001** | 3.04(2.58 to 3.58) | **<0**.**001** |
| **2009-2011 only** |  |  |  |  |  |  |
| Smoking |  |  |  |  |  |  |
| *Non-smoker* | Reference | **-** |  |  |  | - |
| *Quit by 20 weeks* | 0.5(0.16 to 1.57) | 0.24 | NI | - | NI | - |
| *Smoking at 20 weeks* | 1.79(1.27 to 2.51) | **0**.**001** | NI | - | NI | - |
| *Not stated* | 2.20(1.12 to 4.31) | **0**.**02** | NI | - | NI | - |
| Antenatal Care Provider |  |  |  |  |  |  |
| *Obstetrician* | Reference | - |  |  | Reference | - |
| *Midwife* | 0.82(0.62 to 1.07) | 0.14 | NI | - | 0.98(0.66 to 1.46) | 0.91 |
| *GP* | 0.70(0.49 to 0.99) | **0.046** | NI | - | 0.88(0.53 to 1.46) | 0.62 |
| *None/Not Stated* | 5.3(2.42 to11.4) | **<0**.**001** | NI | - | 8.28(2.97 to 23.1) | **<0**.**001** |
| Body Mass Index |  |  |  |  |  |  |
| *<18.5* | 0.95(0.50 to1.78) | 0.86 | NI | - | NI | - |
| *18.5-24.99* | Reference | - | NI | - | NI | - |
| *25-29.99* | 1.23(0.64 to 2.39) | 0.54 | NI | - | NI | - |
| *30-34.99* | 1.16(0.57 to 2.34) | 0.72 | NI | - | NI | - |
| *35plus* | 0.64(0.35 to 1.20) | 0.68 | NI | - | NI | - |

**^1^**Odds Ratio of Stillbirth for each respective variable adjusted for maternal age, maternal region of birth, IRSD, parity 1^st^ trimester ultrasound, pre-existing hypertension, gestational hypertension, APH, detection of SGA, previous stillbirth, GDM, PE/HELLP smoking.

**^2^**Odds Ratio of Stillbirth for each respective variable adjusted for maternal age, maternal region of birth, IRSD, 1^st^ trimester ultrasound, previous stillbirth, gestational hypertension, GDM, PE/HELLP , APH and SGA detection

**^3^**Odds Ratio of Stillbirth for each respective variable adjusted for maternal age, maternal region of birth,parity, IRSD, 1^st^ trimester ultrasound, pre-existing hypertension, gestational hypertension, APH, and SGA detection.

NI- Not included in multivariate model

NB: From 2009 onwards BMI, lead antenatal care provider (obstetrician, midwife, general practitioner, none/not recorded) were available, therefore odds ratios for these variables only apply to years 2009-2011.
